# Effect of A Very Low-Calorie Ketogenic Diet on Food and Alcohol Cravings, Physical and Sexual Activity, Sleep Disturbances, and Quality of Life in Obese Patients

**DOI:** 10.3390/nu10101348

**Published:** 2018-09-21

**Authors:** Ana I. Castro, Diego Gomez-Arbelaez, Ana B. Crujeiras, Roser Granero, Zaida Aguera, Susana Jimenez-Murcia, Ignacio Sajoux, Patricio Lopez-Jaramillo, Fernando Fernandez-Aranda, Felipe F. Casanueva

**Affiliations:** 1 Division of Endocrinology, Department of Medicine, Molecular and Cellular Endocrinology Area, Complejo Hospitalario Universitario de Santiago (CHUS), Instituto de Investigación Sanitaria de Santiago (IDIS), and Santiago de Compostela University (USC), Santiago de Compostela 15706, Spain; anaisabel0121@gmail.com (A.I.C.); diedgomez@gmail.com (D.G.-A.); anabelecrujeiras@hotmail.com (A.B.C.); 2 CIBER de Fisiopatologia de la Obesidad y Nutricion (CIBERobn), Instituto Salud Carlos III, Madrid 28029, Spain; Roser.Granero@uab.cat (R.G.); zaguera@bellvitgehospital.cat (Z.A.); sjimenez@bellvitgehospital.cat (S.J.-M.); ffernandez@bellvitgehospital.cat (F.F.-A.); 3 Faculty of Health Sciences, University of Santander (UDES), Bucaramanga 680003, Colombia; 4 Department of Psychobiology and Methodology, Autonomous University of Barcelona, Barcelona 08193, Spain; 5 Department of Psychiatry, Bellvitge University Hospital-IDIBELL, Barcelona 08908, Spain; 6 Medical Department Pronokal, Pronokal Group, Barcelona 08009, Spain; Ignacio.S@pronokal.com; 7 Center for Research in Metabolic Syndrome, Prediabetes and Diabetes, Fundacion Oftalmologica de Santander (FOSCAL), Floridablanca 681004, Colombia; jplopezj@gmail.com

**Keywords:** ketogenic diet, very low-energy diet, PNK method, protein diet, obesity, food addition, sleep quality, sexual function, QoL

## Abstract

Psychological well-being and hunger and food control are two relevant factors involved in the success of weight-loss therapy in treating obesity. Thus, this study aims to evaluate food and alcohol cravings, physical and sexual activity, sleep, and life quality (QoL) in obese patients following a very low-calorie ketogenic (VLCK) diet, as well as the role of weight lost and ketosis on these parameters. A battery of psychological test was performed in twenty obese patients (12 females, 47.2 ± 10.2 year and BMI of 35.5 ± 4.4) through the course of a 4-month VLCK diet on four subsequent visits: baseline, maximum ketosis, reduced ketosis, and endpoint. Each subject acted as their own control. Relevantly, the dietary-induced changes in body composition (7.7 units of BMI lost, 18 kg of fat mass (1.2 kg of visceral fat mass)) were associated with a statistically significant improvement in food craving scores, physical activity, sleepiness, and female sexual function. Overall, these results also translated in a notable enhancement in QoL of the treated obese patients. Therefore, the rapid and sustained weight and fat mass (FM) loss induced by the VLCK diet is associated with good food control and improvements in the psychological well-being parameters in obese subjects, which could contribute to the long-term success of this therapy.

## 1. Introduction

Obesity is a preventable disease that plagues all the countries of the world, affecting 650 million of its habitants [1,2,3]. In the fight against obesity, a very low-calorie ketogenic (VLCK) diet has consistently been shown to be a useful tool [4,5,6]. Indeed, recent studies from our research group demonstrated that a VLCK diet induces more weight loss than a standard low-calorie diet after 1 and 2 years of follow-up [6]. Among the beneficial effects, the VLCK diet was able to preserve muscle mass, muscle strength [4] and resting metabolic rate [7] and the weight loss was due to fat mass and visceral fat mass [4]. However, obesity is more than an excess body-weight problem [8]. This metabolic disorder is associated with several co-diseases, such as type 2 diabetes, cardiovascular and neurological disorders, and cancer [9,10,11]. Moreover, overweight/obese subjects usually experience a strong desire to eat coupled with a lack of control over eating [12] and suffer from emotional distress and a decrease in quality of life (QoL) and sleep and sexual function [11]. In this regard, few studies have evaluated the effect of weight-loss therapy on the food craving and well-being parameters in overweight/obese subjects. 

Regarding VLCK diets, there is still some distrust in their usage, despite the solid scientific evidence that supports the use of this kind of diet as a useful weight-loss therapy [13,14]. This type of diet is characterized by the restriction of carbohydrates and/or caloric intake to the point of inducing a shift in metabolism and the production of plasma ketone bodies [15,16,17]. However, it was evidenced that a VLCK diet is safe because it does not modify the acid-base equilibrium [18]. In fact, it has been proposed that the ketone body, β-hydroxy-butyrate (β-OHB) has several beneficial effects on metabolism [19] and psychological functions [20,21]. Nevertheless, a scarcity of studies has evaluated the effect of a VLCK diet combined with physical activity, on potential improvement in the psychological conditions and well-being of treated overweight/obese patients. 

In this regard, the aim of this study was to evaluate food and alcohol cravings, physical activity, sleep quality, sexual function, and QoL in patients with obesity after following a VLCK diet. A secondary aim was to elucidate the weight lost and ketosis status effect on the psychological well-being of these patients. 

## 2. Materials and Methods

### 2.1. Cohort of Patients

This 4-month nutritional intervention study was performed in patients attending the Obesity Unit at the Complejo Hospitalario Universitario of Santiago de Compostela (CHUS), Spain. 

The inclusion criteria were age 18 to 65 years, body mass index (BMI) ≥ 30 kg/m^2^, stable body weight in the previous 3 months, a desire to lose weight, and a history of failed dietary efforts. The main exclusion criteria were thyroid alteration, diabetes mellitus, obesity induced by other endocrine disorders or by drugs, and participation in any active weight-loss program in the previous 3 months. In addition, those patients with previous bariatric surgery, known or suspected abuse of narcotics or alcohol, severe depression or any other psychiatric disease, severe hepatic insufficiency, any type of renal insufficiency or gouts episodes, nephrolithiasis, neoplasia, previous events of cardiovascular or cerebrovascular disease, uncontrolled hypertension, orthostatic hypotension, and hydroelectrolytic or electrocardiographic alterations were excluded. Females who were pregnant, breastfeeding, or intending to become pregnant and those with child-bearing potential and not using adequate contraceptive methods were also excluded. Apart from obesity and metabolic syndrome, participants were generally healthy individuals. Under these conditions, 20 obese patients were included in this study.

The study protocol was in accordance with the Declaration of Helsinki and was approved by the Ethics Committee for Clinical Research of Galicia, Santiago de Compostela, Spain (registry 2010/119). Participants gave informed consent before any intervention related to the study. Participants received no monetary incentive.

### 2.2. Very-Low-Calorie Ketogenic Diet Protocol

The nutritional intervention was based on a commercial weight-loss program (PNK method^®^), as was described elsewhere [4]. Briefly, the intervention included an evaluation by the specialist physician conducting the study, an assessment by an expert dietician, and exercise recommendations. This method is based on high-biological-value protein preparations obtained from cow’s milk, soy, avian eggs, green peas, and cereals. Each protein preparation contained 15 g protein, 4 g carbohydrates, 3 g fat, and 50 mg docohexaenoic acid and provided 90–100 kcal.

The weight-loss program has five steps and adheres to the most recent guidelines of the 2015 EFSA on total carbohydrate intake [22]. The first three steps consist of a VLCK diet (600–800 kcal/day), low in carbohydrates (<50 g daily from vegetables), and lipids (only 10 g of olive oil per day). The amount of high biological-value proteins ranged between 0.8 and 1.2 g per each kg of ideal body weight to ensure that patients were meeting their minimum body requirements and to prevent the loss of lean mass. In step 1, the patients ate high-biological-value protein preparations five times a day and vegetables with low glycemic indexes. In step 2, one of the protein servings was substituted with a natural protein (e.g., meat or fish) either at lunch or at dinner. In step 3, a second serving of low-fat natural protein was substituted for the second serving of biological protein preparation. Throughout these ketogenic phases, supplements of vitamins and minerals, such as K, Na, Mg, Ca, and omega-3 fatty acids, were provided in accordance with international recommendations [23]. These three steps were maintained until the patient lost the target amount of weight, ideally 80%. Hence, the ketogenic steps were variable in time depending on the individual and the weight-loss target. The total ketosis state lasted for 60–90 days only.

In steps 4 or 5, the ketogenic phases were ended by the physician in charge of the patient based on the amount of weight lost, and the patient started a low-calorie diet (800–1500 kcal/day). At this point, the patients underwent a progressive incorporation of different food groups and participated in a program of alimentary re-education to guarantee the long-term maintenance of the weight loss. The maintenance diet consisted of an eating plan balanced in carbohydrates, protein, and fat. Depending on the individual, the calories consumed ranged between 1500 and 2000 kcal/day, and the target was to maintain the weight lost and promote a healthy lifestyle.

During this study, the patients followed the different steps of the method until they reached the target weight or up to a maximum of 4 months of follow-up, although patients remained under medical supervision for the following months. Patients visited the research team every 15 ± 2 days to control adherence and evaluate potential side effects. Complete anthropometric, body composition, biochemical and phycological assessments were performed at four of the visits which were made according to the evolution of each patient through the steps of ketosis and weight loss: Visit 1 (baseline), visit 2 (maximum ketosis), visit 3 (reduced ketosis) and visit 4 (Endpoint).

In all the visits, patients received dietary instructions, individual supportive counsel, and encouragement to exercise on a regular basis using a formal exercise program. Additionally, a program of telephone reinforcement calls was instituted, and a phone number was provided to all participants to address any concerns.

### 2.3. Anthropometric Assessment

All anthropometric measurements were undertaken after an overnight fast (8 to 10 h), under resting conditions, in duplicate, and performed by well-trained health workers. Participants’ body weights were measured to the nearest 0.1 kg on the same calibrated electronic device (Seca 220 scale, Medical Resources, EPI Inc. (Lewis Center, OH, USA) in underwear and without shoes. BMI was calculated by dividing body weight in kilograms by the square of height in meters; BMI = weight (kg)/height^2^ (m). 

### 2.4. Total Body Composition 

Body composition was first measured by dual-energy X-ray absorptiometry (DXA; GE Healthcare Lunar, Madison, WI, USA). Daily quality control scans were acquired during the study period. No hardware or software changes were made during the trial. Subjects were scanned using standard imaging and positioning protocols, while wearing only light clothing. For this study, the values of bone mineral density, lean body mass, and FM were directly measured by the GE Lunar Body Composition Software option. Some derivative values, such as bone mineral content, regional lean mass, FFM, fat mass percentage (FM%), and visceral fat mass, were also calculated.

### 2.5. Determination of Levels of Ketone Bodies and Biochemical Parameters

Ketosis was determined by measuring ketone bodies, specifically β-hydroxy-butyrate (β-OHB), in capillary blood by using a portable meter (GlucoMen LX Sensor, A. Menarini Diagnostics, Neuss, Germany; sensitivity <0.2 mmol/L). As with anthropometric assessments, all the determinations of capillary ketonemia were made after an overnight fast of 8 to 10 h. These measurements were performed daily by each patient during the entire VLCK diet, and the corresponding values were reviewed on the machine memory by the research team to control adherence. Additionally, β-OHB levels were determined at each visit by the physician in charge of the patient. Glucose, insulin, HbA1C were performed using an automated chemistry analyzer (Dimension EXL with LM Integrated Chemistry System, Siemens Medical Solutions Inc. (Tarrytown, NY, USA). Thyroid-stimulating hormone (TSH), free thyroxine (FT4), and free triiodothyronine (FT3) were measured by chemiluminescence using ADVIA Centaur (Bayer Diagnostics, Tarrytown, NY, USA). The overnight fasting plasma levels of ghrelin and leptin were measured using commercially available ELISA kits (Millipore, Burlington, MA, USA). The fasting plasma levels of dopamine was tested by high pressure liquid chromatography (HPLC; Reference Laboratory, Barcelona, Spain).

### 2.6. Assessment of Food Cravings and Psychological Well-Being by Self-Questionnaires

Patients were invited to complete a battery of psychological tests to assess performance in the domains of food cravings, quality of life (QoL), daytime sleepiness and sleep quality, sexual functioning, and physical activity through the course of the nutritional intervention. The psychological tests were selected for availability of multiple test versions, well-stablished psychometric properties, and accepted clinical use.

### 2.7. Food Craving Questionnaires 

Food craving refers to an intense desire to consume a specific food. The food cravings questionnaires (FCQs) [24] assess food cravings on a trait and a state level and on a specific food item. The FCQ-trait was derived from a total of 88 statements that were generated using 10 theoretical dimensions of trait food cravings. Participants were asked to indicate how frequently each statement “would be true for you in general” using a 6-point scale that ranged from “Never” or “Not Applicable” to “Always”. The FCQ-state was derived from a total of 60 statements representing seven dimensions of state food cravings. Participants were asked to indicate the extent to which they agreed with each statement “right now, at this very moment” using a Likert scale that ranged from “Strongly Agree” to “Strongly Disagree”. 

The FCQ-inventory was based on the validated FCQ-inventory containing 28 item foods. Participants were instructed to indicate how often, in the last month, they have experienced food cravings for each item on a Likert scale where 1 = never, 2 = rarely, 3 = sometimes, 4 = often, and 5 = always/almost every day. There were 3 subscales that categorized foods of similar composition: simple sugars/trans fats, complex carbohydrate/proteins, and saturated fats/high caloric content. To calculate each subscale score, the values given for the corresponding items were summed, and the mean was recorded. A higher score in the FCQ indicated greater food cravings.

Alcohol craving was evaluated by using the multidimensional alcohol craving scale (MACS), which measures desire and behavioral disinhibition (lack of resistance), and it was entirely developed in Spain and consists of 12 items [25]. 

### 2.8. Physical Activity 

Physical activity was determined by means of the International Physical Activity Questionnaire (IPAQ), which is used worldwide to indirectly evaluate an individual’s volumes of sedentary behavior and moderate to vigorous physical activity throughout the last seven-day week [26]. It is readily available, very cost-effective, and can generate large data sets. The IPAQ comprises a set of 4 questionnaires. In this study, the short form of the IPAQ was employed (7 items). This measure assesses the types of intensity of physical activity and sitting time that people do as part of their daily lives, which is used to estimate total physical activity in metabolic equivalent task minutes per week (MET-min/week) and time spent sitting [26]. 

### 2.9. Sexual Functionality Questionnaire

Sexual functioning in men was explored by means of the EMAS-Sexual Function Questionnaire (EMAS-SFQ) [27]. This questionnaire was completed in private and then placed in a sealed envelope by the participants without scrutiny by the researchers. The EMAS-SFQ has been found to exhibit excellent internal and test-retest reliability and convergent, divergent, and discriminant validity in psychometric analyses [27]. It consists of 16 items assessing sexual functioning, distress or worry relating to current functioning, and changes in sexual functioning compared with 1 year ago. 

Women were also invited to complete a questionnaire on sexual function (the Female Sexual Function Index—FSFI). The FSFI consists of 19 questions, divided into 6 domains: desire, arousal, lubrication, orgasm, satisfaction, and pain. Each answer is rated on a scale ranging from 0 to 5 or 1 to 5 (0 means no sexual activity in the four preceding weeks) [28]. The total FSFI score, obtained from the sum of the items in each domain multiplied by the domain factor (0.6 for desire, 0.3 for arousal and lubrication, and 0.4 for orgasm, satisfaction, and pain), may range from 2.0 to 36.0. Lower scores indicate poorer sexual function. A total FSFI score less than 26.55 is indicative of sexual dysfunction [28]. 

### 2.10. Epworth Daytime Sleepiness Scale (ESS)

The ESS is based on questions referring to eight such situations, some known to be very soporific and others less so. The questionnaire is self-administered, and the item scores provide a new method for measuring sleep propensity in eight different real-life situations. Subjects are asked to rate on a scale of 0–3 how likely they would be to doze off or fall asleep in the eight situations, based on their usual, current lifestyle. A distinction is made between dozing off and simply feeling tired. If a subject has not been in some of the situations recently, he or she is asked, nonetheless, to estimate how each might affect him or her [29]. 

### 2.11. Pittsburgh Sleep Quality Index (PSQI)

The PSQI questionnaire is a clinical sleep-behavior questionnaire that has been validated for use in patients with insomnia, cancer, Parkinson’s disease, and the general population [30]. The questionnaire is designed to assess indexes of sleep during the preceding month and contains 19 questions that use Likert scales from 0–3. All questions are categorized into the following 7 subvariables: duration of sleep, sleep disturbance, sleep latency, day dysfunction because of sleepiness, sleep efficiency, subjective sleep quality, and use of a sleeping medication. Each of these 7 variables is scored between 0 and 3 arbitrary units (au), which generates a summed total score of 0–21 au. This total score is termed the global sleep score (GSS) with >5 au associated with a poor sleep condition and ≤5 au associated with a good sleep condition. 

### 2.12. Impact of Weight on Quality of Life-Lite (IWQOL-LITE)

QoL was evaluated by the Impact of Weight on QoL (IWQOL-Lite©) questionnaire [31], a 31-item questionnaire evaluating social, professional, and sexual life, self-esteem, and mobility. Higher scores signal a better QoL in the domain evaluated. 

The IWQOL-Lite, a shorter form of the original questionnaire, assesses the impact of weight on quality of life in individuals exploring treatments for weight loss. The IWQOL-Lite includes 31 statements that start with “Because of my weight…” with response options to each statement ranging from (1) “Never true” to (5) “Always true” that measure the impact of weight on 5 domains (i.e., physical function, self-esteem, sexual life, public distress, and work life). A score is calculated for each domain for each patient who answers at least 50% of the questions in any given domain. A total score is calculated if patients have responded to at least 26 out of the 31 questions. The total score is the sum of the raw scores of the 5 subscales. Raw scores (higher scores indicate poorer quality of life on the IWQOL-Lite) are converted into a T-score (0–100), with 100 representing the best possible health. Mean and standard deviations are reported for each domain [32].

### 2.13. Statistical Analysis

The statistical analysis was carried out with Stata15 for Windows. The estimated sample size was based on a repeated-measures ANOVA design with a 4-level within-subject factor, taking into account weight loss after treatment and defining the next parameters: a potential correlation of ρ = 0.30 between measurements, maximum error variance σ = 300 (this value is based on the results published for the type of measures analyzed in the study), default power of 1-β = 80%, alpha level α = 5%, and delta of at least δ = 0.772 (it corresponds to an effect size between the baseline and final measurement of at least 15 kg). The sample size estimated a minimum of *n* = 20 participants.

The changes in the variables of the study were analyzed through repeated-measures ANOVA (the within-subjects factor included 4 measurements), defining polynomial contrast to explore potential linear, quadratic, and cubic trends (3-order polynomial trends were considered due to the 4-level measurements), and post-hoc pairwise comparisons employing Tukey’s adjustment for multiple comparisons. Effect size for each pairwise comparison was based on Cohen’s-*d* coefficient, considering poor effect size for |*d*| > 0.20, medium/moderate for |*d*| > 0.50, and large/good for |*d*| > 0.80 [33]. 

The association between the physical and psychological changes in the measures of the study was estimated with bivariate Pearson’s correlation. Due to the strong dependence, the relevance of these coefficients was not based on a significance test (low samples sizes tended to report nonsignificant R-coefficients, which effect size could be considered high), effect size was considered poor for |*R*| > 0.10, medium for |*R*| > 0.24, and large for |*R*| > 0.37 (these thresholds correspond to Cohen’s-*d* of 0.20, 0.50 and 0.80, respectively) [34]. 

## 3. Results 

### 3.1. Changes in Anthropometrical and Body Composition Measurements

Twenty obese patients, 12 females, aged from 18 to 58 years (47.2 ± 10.2 year) and BMI of 35.5 ± 4.4, completed the study (Table 1). Other baseline characteristics and their corresponding changes during the study have been previously reported [4]. 

**Figure 1:**
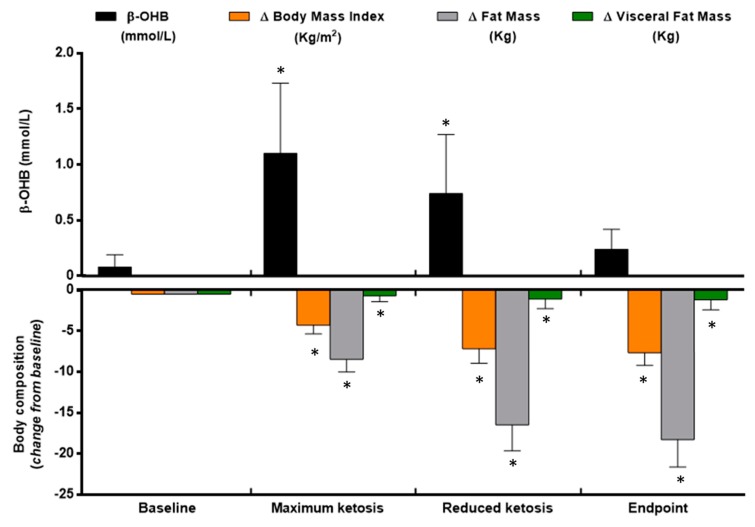
Ketone bodies and body composition changes during the very low-calorie-ketogenic diet treatment. Data represent mean ± standard error of changes from baseline. (*) Denotes statistically significant differences as compared with baseline. (△) Denotes differences from baseline. β-OHB, β-Hydroxy-Butyrate.

By design, the nutritional intervention induced an important reduction in BMI and fat mass, especially visceral fat mass through the study visits synchronized with the ketone levels in four visits (Figure 1). Thus, at the end of the nutritional intervention, the patients were out of ketosis (0.2 ± 0.1 mmol/L) with a total of 7.7 units of BMI lost (Figure 1). Most of the initial body composition loss was in the form of total fat mass (Figure 1). Relevantly, from the total fat mass, visceral fat mass, the most physiological and clinically relevant fat depot, was significantly reduced after the VLCK diet (−1.2 ± 0.7 kg; *p* < 0.05). 

### 3.2. Changes in Craving and Well-Being Self-Reported Test

#### 3.2.1. Effect of the Nutritional Intervention on Food and Alcohol Cravings

**Figure 2:**
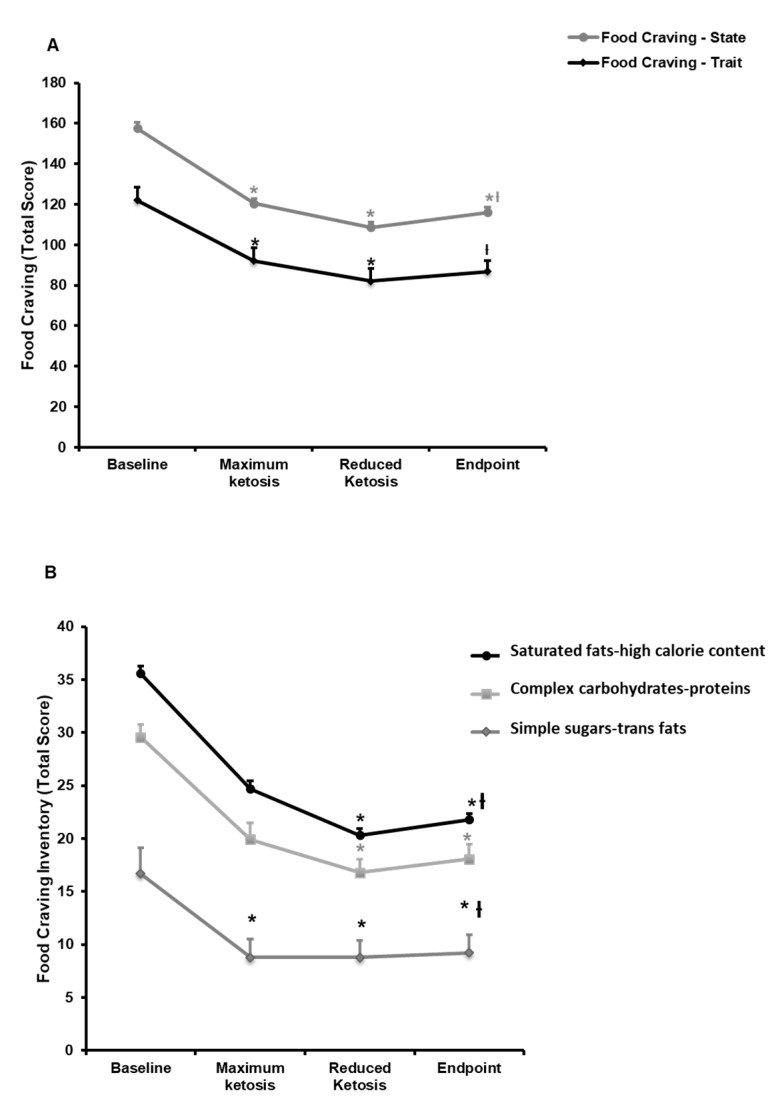
Changes in food craving during the very low-calorie-ketogenic diet treatment. (**A**) Food craving trait and state. (**B**) Food craving inventory. Data represent mean ± standard error of changes from baseline. (ƚ) Denotes statistically significant differences through the intervention (*p* for trend < 0.05) evaluated by means of repeated-measures ANOVA. (*) Denotes statistically significant differences (*p* < 0.05) from baseline after post-hoc pairwise comparisons employing the Tukey’s adjustment for multiple comparisons.

Questionnaires were used to evaluate food craving as a trait, as a state, and to different nutrients. Statistically significant decreases were observed in the global score of trait and state when comparing all visits with baseline (Figure 2A). More specifically, the eight items of the FCQ-T (Table S1) decreased with statistical significances since the visit of maximum ketosis, except for the positive and negative reinforcement, which exhibited differences since the visit of reduced ketosis. Relevantly, a negative correlation was observed between B-OHB levels and the intention to eat (*r* = −0.46; *p* < 0.05) and feelings of hunger (*r* = −0.30; *p* < 0.05) during the phase of maximum ketosis. However, these effects on feelings of hunger were not evidenced at circulating levels of ghrelin, which showed no statistically significant changes during the intervention (data not shown).

Regarding the FCQ-S, the total state score decreases significantly when compared with the visit of maximum ketosis and the visit of reduced ketosis with baseline; but no statistically significant differences were observed between endpoint and baseline. 

Relevantly, a statistically significant decrease was also observed in the craving for specific nutrients from baseline to endpoint (Figure 2B). These modifications in FC-inventory were evidenced from the visit of reduced ketosis as compared with baseline. Whereas, the craving for simple sugars and trans fats was modified earlier than the other items, since maximum ketosis compared with baseline (Figure 2B).

When the craving for alcohol was evaluated, no statistically significant changes were observed in the MACS scores through the nutritional intervention, taking all patients together (Table S1). However, when the analysis was performed considering the gender of participants in the study, men experienced a significant decrease in the total score through the study (*p* = 0.047). This decrease was more notable in the maximum ketosis phase as compared with baseline (−15.14; *p* = 0.047). Moreover, a statistically significant reduction was observed in the lack of inhibition item (−27.19; *p* = 0.042).

#### 3.2.2. Effect of the Nutritional Intervention on Physical Activity 

**Figure 3:**
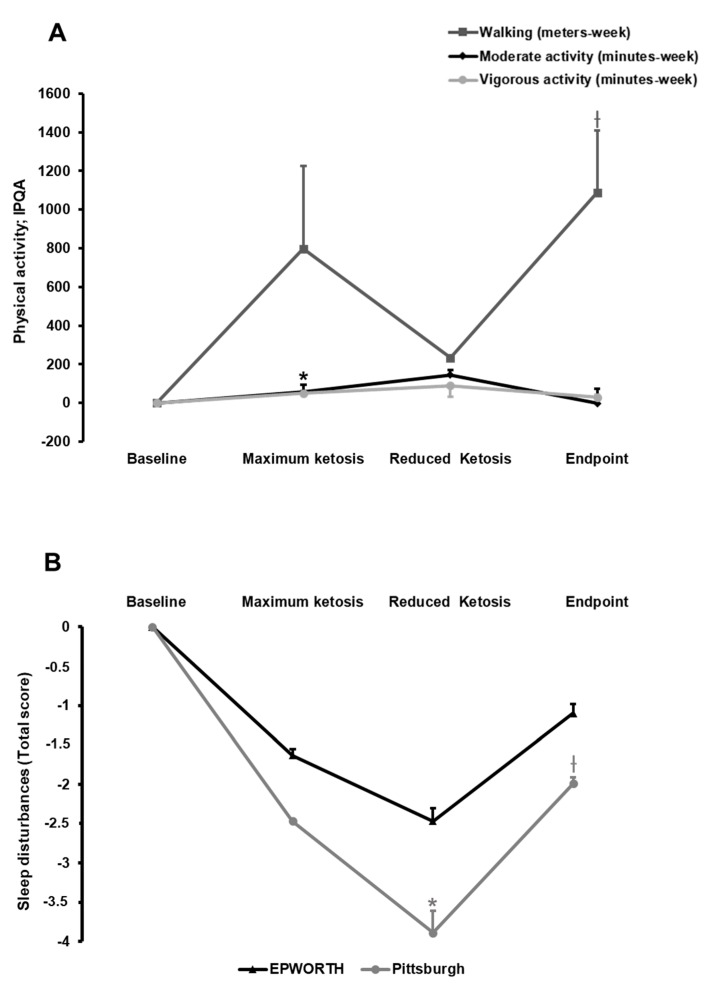
Effect of the nutritional intervention with a very low-calorie-ketogenic diet on physical activity pattern (**A**) and sleep disturbances (**B**). Data represent mean ± standard error of changes from baseline. (ƚ) Denotes statistically significant differences through the intervention (*p* for trend < 0.05) evaluated by means of repeated-measures ANOVA. (*) Denotes statistically significant differences (*p* < 0.05) from baseline after post-hoc pairwise comparisons employing the Tukey’s adjustment for multiple comparisons. IPQA, International Physical Activity Questionnaire.

During the nutritional intervention, a trend for increasing physical activity was observed, especially in the walking domain (Figure 3A). Patients increased their walking distance for a week (*p* = 0.05). Moreover, patients increased their vigorous activity during the maximum ketosis phase in 100 min/week when compared to baseline (*p* = 0.032).

#### 3.2.3. Effect of the Nutritional Intervention on Sleep Quality

Sleep quality was evaluated by determining sleep propensity and quality by means of the PSS and PSQI, respectively. Overall, participants showed a poor sleep condition with a total score >5 au (Table S1). Thus, a significant improvement in sleepiness (PSS) was observed when comparing the visit of reduced ketosis with baseline, a point that coincided with maximum loss of fat mass (Figure 3B). By contrast, no statistically significant effect was observed on sleep quality and duration (PSQI; Figure 3B). Accordingly, plasma levels of dopamine showed no statistically significant changes (data not shown).

#### 3.2.4. Effect of the Nutritional Intervention on Sexual Function

**Figure 4:**
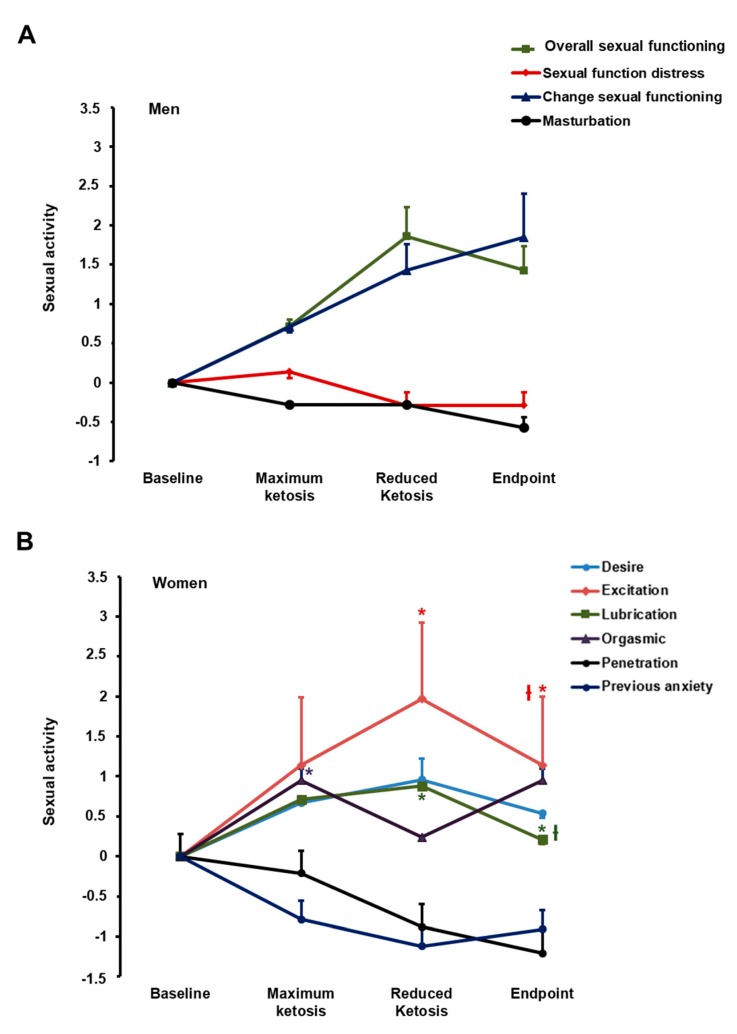
Effect of the nutritional intervention on sexual activity in men (**A**) and women (**B**). Data represent mean ± standard error of changes from baseline. (ƚ) Denotes statistically significant differences through the intervention (*p* for trend < 0.05) evaluated by means of repeated-measures ANOVA. (*) Denotes statistically significant differences (*p* < 0.05) from baseline after post-hoc pairwise comparisons employing the Tukey’s adjustment for multiple comparisons.

An interesting effect on sexual function was induced by the nutritional intervention (Table S1; Figure 4). The EMAS-SF questionnaire reported no statistically significant changes for sexual activity in men (Figure 4A). However, the FSFI questionnaire for sexual activity in women evidenced that excitation (*p* = 0.043) and lubrication (*p* = 0.013) improved with statistical significance throughout the study. Moreover, from baseline to maximum ketosis, a statistically significant increase was observed in the score for the orgasmic domain (Figure 4B; 0.95; *p* = 0.034). Based on the FSFI mean total score, women included in this study showed sexual dysfunction (total score = 9.55) at baseline. This total score was improved at maximum of ketosis (total score = 10.48) and at the end of the nutritional intervention (total score = 9.8). 

#### 3.2.5. Effect of the Nutritional Intervention on the Impact of Weight on Quality of Life-Lite (IWQOL-Lite)

During the ketosis phase of the nutritional intervention, the IWQOL-Lite scores did not change for the sexual life, social anxiety, and work area domains (Table S1). A significant improvement was observed in the physical function and self-esteem scores during this phase. When comparing the visit of reduced ketosis and endpoint with baseline, a significant improvement was found in all domains, except for social anxiety, which did not change throughout the nutritional intervention. 

**Figure 5:**
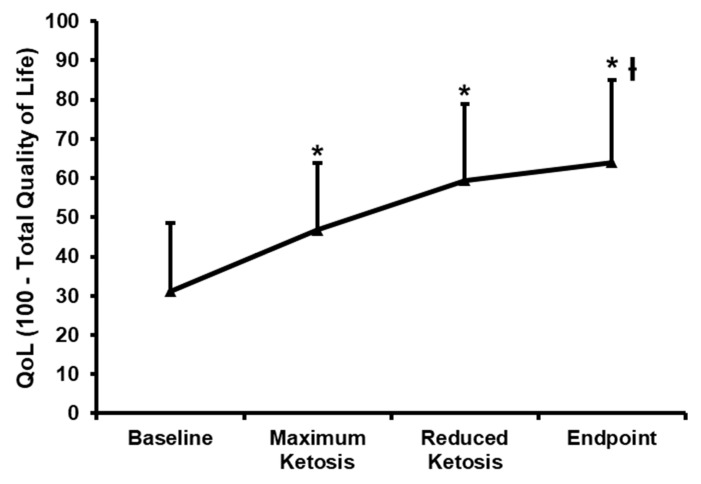
Changes in the impact of weight on quality of life-lite (IWQOL-Lite). Raw scores (higher scores indicate poorer quality of life on the IWQOL-Lite) were converted into a *T*-score (0–100), with 100 representing best possible health. Mean ± standard error of changes from baseline are reported for total score. (ƚ) Denotes statistically significant differences through the intervention (*p* for trend < 0.05) evaluated by means of repeated-measures ANOVA. (*) Denotes statistically significant differences (*p* < 0.05) from baseline after post-hoc pairwise comparisons employing the Tukey’s adjustment for multiple comparisons.

Relevantly, the IWQOL-Lite total score was significantly lower in baseline than the other points of the nutritional intervention (31.1; 46.8; 59.5 and 64.1; *p* < 0.01; Figure 5). 

### 3.3. Association between the Physical and Psychological Changes during the Follow-Up

Table 2 contains the correlation matrix that assessed the association between the changes in the physical and psychological states. Many relevant *R*-coefficients were found, confirming that changes in body composition (BMI, FM, FFM, and weight) and ketosis were strongly related to the differences in food craving, alcohol craving, sleep patterns, physical activity, and sexual activity. These associations emerged when changes between baseline and maximum ketosis were assessed, and they remained quite similar for changes estimated between baseline and reduced ketosis or endpoint. Regarding the ketosis change levels, associations emerged with some subscales in all the psychological questionnaires, except for the FCI scales and the multidimensional alcohol craving measures. 

## 4. Discussion

This work demonstrates that a VLCK diet following the PNK method induces a severe body weight reduction concomitantly with a decrease in food craving and improvements in psychological well-being measured by physical activity, sleep quality, female sexual function, and quality of life scores. The effect in food craving and psychological well-being could be relevant factors to guarantee the success of this kind of nutritional treatment. Moreover, this effect is added to the beneficial effects previously observed regarding body composition, energy metabolism, and biochemical parameters [4,7,18,35].

Despite the efforts to decrease weight loss, obesity prevalence is increasing worldwide [36]. The obesogenic environment and the unsuccessful effect of current treatments are consistently contributing to an increase in obesity prevalence. Obesity is promoted by several factors, including genetic, environmental, metabolic, and behavioral factors [8,11,37]. These same factors are involved in the unsuccessful effect of many weight-loss therapies [38]. Apart from the biochemical and genetic factors, in the literature, obesity has consistently been related with a poorer quality of life [39] and lower self-esteem and lower life satisfaction [40]. Additionally, food addiction was proposed as a plausible causal factor contributing to obesity and weight regain after a weight-loss therapy, at least in the same individuals [41]. Therefore, it is important to control these factors to attain success in weight-loss therapy. In this context, a VLCK diet has previously been shown to induce severe body-weight loss that has been maintained for at least 2 years after dieting [6]. This nutritional weight-loss method resulted in the beneficial effects of decreasing body fat mass by preserving body muscle mass and strength [4] and maintaining the resting metabolic rate [7]. Thus, the new open question was whether the beneficial effects of this nutritional method on body composition and energy metabolism are associated with a modulation in the psychobiological phenomena of obese patients. 

For obesity-reduction experts, it is well known that the main obstacle to follow a hypocaloric diet is hunger. In fact, within a few days after undertaking such a calorie-lowered diet, patients suffered a battery of negative effects, such as hunger, sadness, bad humor, and, in some cases, mild depression. All these side effects were absent in the patients following a VLCK diet, thus contributing to the success of these types of treatments. The mechanism that erases hunger and sadness in obese subjects following a VLCK diet are not known, and several authors strongly believe that it is due to the anorexigenic effect of ketosis [42]. As a result, of that rationale, the target of this work was to study the neurocognitive effects of ketosis, using a battery of neurocognitive and QoL tests in the same individuals at three different stages; (a) nonketosis-nonweight reduction (basal), (b) highly ketosis-mild weight reduction (visit 2), and (c) nonketosis-strong (mean 20 kg) weight reduction. 

Because the weight-loss method employed in this study consisted of a reduction of energy intake to less than 800 kcal/day in the first stage of the treatment, it could be expected that there would be an increase in food craving in response to metabolic need expressed as hunger. By contrast, in this study, a reduction in food craving was observed despite the high energy restriction. These results agreed with previous studies that also concluded a reduced food craving after an energy restriction diet [43]. In fact, it has been demonstrated that energy restriction via a liquid formula-based total meal replacement very low-calorie diet suppresses food cravings compared to energy restriction via a typical food-based low-calorie diet [44]. This decrease in food craving associated with energy restriction was recently demonstrated to be consistent with increased executive control over ingestion and food cravings, by examining human brain functional MRI food-cue reactivity (fMRI-FCR) [45]. As in that study, we observed a significant reduction of overall food cravings and cravings for sweet food, high-fat food, starchy food, and fast food as measured by the food craving inventory questionnaire [45] after the VLCK diet. Relevantly, when we focused on specific subscales of the food-craving questionnaire, we observed that high levels of ketone bodies correlated with low scores of hunger feelings and intentions to eat. These results are in line with the effect of ketosis on food control previously reported [14,46,47]. However, contrary to a previous study, which demonstrates a lowering of plasma ghrelin levels induced by ketone ester drinks [48], in the current work, the circulating levels of ghrelin were not modified despite the increase in blood ketone levels (data not shown). Additionally, the VLCK diet-PNK method was able to maintain the reduction in hunger during the intervention, even at the no ketosis phase, in contrast with a previous work that evidenced an increase in hunger during the refeeding phase [49].

Another concern for obese individuals is physical activity and sexual functioning. Relevantly, an increase in physical activity was observed during the intervention. This improvement agrees with the design of the nutritional intervention because the commercial weight-loss program (PNK method) includes lifestyle and behavioral modification support. All patients underwent a structured program of physical exercise with external supervision. According to these results, patients are encouraged to walk and to practice vigorous exercise. 

Sexual dysfunction in subjects with obesity has been scarcely studied. Recent studies evidenced a rate of sexual dysfunction in 29–60% of women and 24.8–45% of men with obesity [50]. In the current study, at baseline, the obese men reported a relatively good sexual function based on the EMAS–SFQ. The domains included in this questionnaire correlated with levels of testosterone, and the EMAS-SFQ scores reported in the current study are higher than those previously observed in patients with low levels of testosterone [27]. Accordingly, we did not find significant changes in the sexual function of men during the nutritional intervention. By contrast, women reported a general sexual dysfunction that improved, especially in the maximum ketosis phase, and then during the intervention. More concretely, women exhibited an improvement in excitation, lubrication, and orgasm capacity. Sexual dysfunction is usually related to impairments in parameters related to healthy, body image dissatisfaction, depressive symptoms, and lower levels of romantic relationship satisfaction [51]. Then, it could be hypothesized that the beneficial effects on several parameters of body composition and biochemicals induced by the VLCK diet, PNK method^®^, could be involved in an improvement in the sexual function of obese women. 

Importantly, in the current study, an improvement in the indexes of sleep was observed, especially at the point of maximum loss of fat mass (reduced ketosis). Sleep is essential to health and is associated with morbidities and mortality associated with obesity [52,53,54,55,56,57]. A recent study demonstrates that dietary protein intake while dieting to lose weight may improve sleep quality in overweight and obese adults [30]. We were unable to detect statistically significant changes in the sleep quality measured by the PSQI and indirectly corroborated with no changes during the treatment in the plasma levels of dopamine, a brain neurotransmitter synthetized from tryptophan. By contrast, the sleepiness during different real-life situations measured by the ESS was significantly reduced. This result is important because it can be associated with an improvement in the QoL, influencing parameters such as physical functioning, sexual life, and work activity. These parameters were improved after the VLCK diet treatment and contributed to an enhanced global score of quality of life evaluated by the IWQoL-Lite. The strength of this study is its longitudinal design, which allows the evaluation of the time-course of changes of psychological well-being induced by a VLCK diet. The small sample size of this study might be a limitation; however, as each subject underwent 4 evaluations and results compare with themselves, this adds statistical power to the study and a real difference between the experimental points. Moreover, the short follow-up of this study (4 months) increases the relevance of the findings as they were strong enough to be evident so rapidly. Other limitation could be that the results of this study were not comparable with a standard nonketogenic low-calorie diet. This was because this analysis was performed in previous studies [58,59,60,61,62]. 

It was recently reported that the consumption of diets with low percentages of carbohydrates over a long period of time (>25 years) are associated with higher mortality [63]. This association was mitigated when the substitutions were plant-based [63]. In the current study, patients were exposed to a low carbohydrate intake during less than 90 days and the source of carbohydrate was from vegetables. Moreover, the strong weight loss induced a decrease in the burden of obesity-related disease [6]. Thus, the strength of the current study is reinforced with the consistence of the results with that of previous research, regardless of the strong dietary energy restrictions induced by a VLCK diet. 

In conclusion, to the best of our knowledge, this is the first study that exhaustively assessed food and alcohol cravings and changes in well-being determinants, such as sexual function, physical activity, and sleep abnormalities and QoL, during the weight-reduction process induced by a VLCK diet. The severe weight loss induced by the VLCK diet-PNK method was concomitant with a decrease in food and alcohol cravings, increases in physical activity, reduction of sleep abnormalities, and improvement in sexual functioning. Overall, these psychobiological parameters were translated to an enhancement in general QoL for the dieters. Relevantly, this is the first study able to distinguish the effect of ketosis per se independently of the weight-loss magnitude because the strongest effect was evidenced at highly ketosis-mild weight reduction, rather than at nonketosis-strong (mean 20 kg) weight reduction. Therefore, the results of this study evidenced that the rapid and sustained weight and FM loss induced by VLCK-diet are associated with good food control and improvements in the psychological well-being parameters in obese subjects that could be reinforced by the effect of ketosis. This effect could contribute to long-term success of this therapy and further reinforce the suitability of a VLCK-diet as a viable and valuable treatment option for obesity. 

## Figures and Tables

**Table 1:** Baseline characteristics of patients.

Variable	Baseline
Age (years)	47.2 (10.2)
Gender (M/F)	8/12
β-Hydroxy-Butyrate (mmol/L)	0.08 (0.1)
Body Mass Index (kg/m^2^)	35.5 (4.4)
Waist Circumference (cm)	109.4 (12.8)
Fat Mass (kg)	42.2 (9.1)
Fat Mass Percentage (%)	45.6 (5.4)
Visceral Fat Mass (kg)	2.18 (1.28)
Fat-Free Mass (kg)	52.8 (10.2)
Bone Mineral Content (kg)	2.59 (0.49)
Bone Mineral Density (g/cm^2^)	1.24 (0.13)
Systolic Blood Pressure (mmHg)	125.6 (12.5)
Diastolic Blood Pressure (mmHg)	86.1 (7.6)
Glucose (mg/dL)	96.0 (11.7)
Insulin (mUI/L)	20.4 (10.7)
HOMA-IR	5.0 (2.8)
HbA1c (%)	5.7 (0.4)
TSH (pg/dL)	1.9 (1.0)
Free T4 (pg/dL)	1.1 (0.1)
Free T3 (ng/dL)	3.4 (0.3)

Data are presented as mean (standard deviation). TSH, Thyroid-Stimulating Hormone.

**Table 2:** Association between the physical and psychological changes: Pearson’s correlations (*n* = 20).

	Change between Assessments 1–2					Change between Assessments 1–3					Change between Assessments 1–4				
	BMI	FM	FFM	Weight	Ketosis	BMI	FM	FFM	Weight	Ketosis	BMI	FM	FFM	Weight	Ketosis
Food Craving Questionnaire (FCQ)-Trait															
Positive Reinforcement	**0.424 ^†^**	**0.380 ^†^**	0.102	**0.392 ^†^**	−0.157	**0.463 ^†^**	**0.358 ^†^**	**0.391 ^†^**	**0.416 ^†^**	−0.136	**0.326 ^†^**	**0.269 ^†^**	**0.371 ^†^**	**0.327 ^†^**	−0.122
Negative Reinforcement	**0.312 ^†^**	0.144	**0.545 ^†^**	**0.334 ^†^**	−0.178	**0.459 ^†^**	**0.265^†^**	**0.489 ^†^**	**0.365 ^†^**	−0.134	**0.408 ^†^**	**0.388 ^†^**	**0.463 ^†^**	**0.456 ^†^**	−0.078
Intentions to Eat	0.177	0.025	**0.465 ^†^**	0.201	**−0.455 ^†^**	**0.317 ^†^**	**0.324 ^†^**	**0.312 ^†^**	**0.371 ^†^**	**−0.293 ^†^**	**0.266 ^†^**	**0.315 ^†^**	**0.278 ^†^**	**0.348 ^†^**	−0.030
Cue-Dependent Eating	0.078	−0.085	**0.297 ^†^**	0.031	−0.195	0.211	−0.005	0.178	0.044	**−0.347 ^†^**	**0.329 ^†^**	0.107	0.206	0.147	**−0.253 ^†^**
Thoughts-Preoccupations with Food	**0.271 ^†^**	0.108	0.234	0.186	−0.056	**0.337 ^†^**	0.229	**0.290 ^†^**	**0.278 ^†^**	−0.052	**0.331 ^†^**	0.206	**0.512 ^†^**	**0.317 ^†^**	−0.027
Feelings of Hunger	0.212	0.110	**0.353 ^†^**	0.235	**−0.303 ^†^**	0.214	0.214	**0.245 ^†^**	**0.258 ^†^**	−0.141	0.206	0.230	**0.250 ^†^**	**0.268 ^†^**	−0.007
Lack of Control	**0.294 ^†^**	−0.007	**0.645 ^†^**	**0.244 ^†^**	−0.149	**0.485 ^†^**	**0.342 ^†^**	**0.393 ^†^**	**0.410 ^†^**	−0.156	**0.546 ^†^**	**0.448 ^†^**	**0.518 ^†^**	**0.524 ^†^**	−0.199
Negative Affect	**0.284 ^†^**	−0.016	**0.483 ^†^**	0.166	−0.129	**0.505 ^†^**	**0.290 ^†^**	**0.473 ^†^**	**0.386 ^†^**	−0.195	**0.352 ^†^**	0.207	**0.281 ^†^**	**0.251 ^†^**	−0.031
Guilty Feelings	0.131	−0.001	0.111	0.029	−0.173	**0.325 ^†^**	0.179	−0.070	0.130	**−0.340 ^†^**	**0.393 ^†^**	**0.243 ^†^**	0.159	**0.246 ^†^**	**−0.256 ^†^**
Total Trait Score	**0.309 ^†^**	0.093	**0.443^†^**	**0.252 ^†^**	−0.229	**0.466 ^†^**	**0.309 ^†^**	**0.376 ^†^**	**0.373 ^†^**	−0.229	**0.464 ^†^**	**0.353 ^†^**	**0.458 ^†^**	**0.426 ^†^**	−0.148
Food Craving Questionnaire (FCQ)-State															
Intense Desire to Eat	0.003	0.007	**−0.299 ^†^**	−0.105	0.077	0.185	0.126	−0.168	0.057	**−0.561 ^†^**	0.236	0.152	**0.288 ^†^**	0.210	**−0.543 ^†^**
Anticipation Positive Reinforcement	**0.331 ^†^**	0.214	**0.285 ^†^**	**0.303 ^†^**	−0.023	0.197	0.114	0.207	0.158	**−0.365 ^†^**	**0.424 ^†^**	**0.380 ^†^**	**0.410 ^†^**	**0.434 ^†^**	**−0.467 ^†^**
Anticipation Relief from Negative States	0.196	0.145	**0.362 ^†^**	**0.266 ^†^**	**−0.287 ^†^**	**0.267 ^†^**	0.234	0.212	**0.262 ^†^**	**−0.294 ^†^**	**0.275 ^†^**	**0.285 ^†^**	0.233	**0.305 ^†^**	−0.143
Preoccupation with Food-Lack Control	0.160	0.011	**0.391 ^†^**	0.154	**−0.415 ^†^**	**0.381 ^†^**	**0.241 ^†^**	**0.408 ^†^**	**0.323 ^†^**	**−0.260 ^†^**	**0.387 ^†^**	**0.314 ^†^**	**0.431 ^†^**	**0.388 ^†^**	−0.049
Craving as Physiological State	**−0.376 ^†^**	−0.195	**−0.368 ^†^**	**−0.325 ^†^**	−0.113	−0.134	−0.021	**−0.313 ^†^**	−0.111	**−0.542 ^†^**	−0.055	−0.031	−0.009	−0.030	−0.083
Total State Score	0.117	0.071	0.114	0.104	−0.208	0.209	0.165	0.063	0.158	**−0.514 ^†^**	**0.349 ^†^**	**0.307 ^†^**	**0.366 ^†^**	**0.361 ^†^**	**−0.353 ^†^**
Food Craving Inventory (FCI, SP)															
Simple Sugars/Trans Fats	**−0.327 ^†^**	**−0.487 ^†^**	0.155	**−0.391 ^†^**	**−0.454 ^†^**	**−0.377 ^†^**	**−0.556 ^†^**	0.174	**−0.446 ^†^**	−0.227	−0.171	**−0.371 ^†^**	0.136	**−0.278 ^†^**	−0.121
Complex Carbohydrates/Proteins	−0.079	−0.203	0.092	−0.160	**−0.420 ^†^**	−0.228	**−0.346 ^†^**	**0.364 ^†^**	−0.207	−0.075	−0.178	**−0.306 ^†^**	**0.317 ^†^**	−0.175	−0.084
Saturated Fat/High Calorie Content	**0.418 ^†^**	0.198	**0.310 ^†^**	**0.281 ^†^**	−0.239	0.190	−0.024	**0.520 ^†^**	0.121	−0.063	0.062	−0.084	**0.326 ^†^**	0.017	0.230
Multidimentsional Alcohol Craving Scale (MACS)															
Desire	−0.058	−0.015	**0.293 ^†^**	0.097	**−0.273 ^†^**	0.135	0.111	**0.284 ^†^**	0.172	0.101	**0.253 ^†^**	0.240	**0.285 ^†^**	**0.280 ^†^**	0.191
Lack of Inhibition	0.138	**0.271 ^†^**	−0.104	0.210	**0.338 ^†^**	0.111	0.188	0.041	0.178	0.029	0.154	0.145	0.158	0.163	0.124
Total Score	0.039	0.166	0.192	0.225	−0.019	0.204	0.231	**0.305 ^†^**	**0.284 ^†^**	0.118	**0.302 ^†^**	**0.285 ^†^**	**0.332 ^†^**	**0.330 ^†^**	0.232
Impact of Weight on Quality of Life-Lite															
Physical	**0.311 ^†^**	**0.327 ^†^**	0.229	**0.384 ^†^**	**0.354 ^†^**	**0.319 ^†^**	**0.426 ^†^**	**0.303 ^†^**	**0.461 ^†^**	**0.422 ^†^**	**0.414 ^†^**	**0.478 ^†^**	**0.360 ^†^**	**0.502 ^†^**	**0.274 ^†^**
Sexual Life	−0.072	−0.177	0.211	−0.078	−0.133	0.159	−0.061	**0.580 ^†^**	0.110	−0.038	**0.379 ^†^**	0.215	**0.525 ^†^**	**0.325 ^†^**	0.039
Self-Steam	−0.201	−0.214	−0.077	−0.224	0.122	−0.155	**−0.266 ^†^**	−0.003	−0.233	−0.083	0.062	−0.108	0.181	−0.045	0.044
Social Anxiety	0.215	0.086	0.235	0.168	0.137	**0.603 ^†^**	**0.347 ^†^**	**0.666 ^†^**	**0.488 ^†^**	**−0.327 ^†^**	**0.640 ^†^**	**0.405 ^†^**	**0.740 ^†^**	**0.540 ^†^**	0.209
Work Area	**0.295 ^†^**	0.068	**0.417 ^†^**	0.205	**0.447 ^†^**	0.197	0.130	0.217	0.177	−0.143	**0.458 ^†^**	**0.313 ^†^**	**0.449 ^†^**	**0.386 ^†^**	0.042
Total Score	0.147	0.086	0.231	0.165	**0.313 ^†^**	**0.330 ^†^**	0.217	**0.520 ^†^**	**0.338 ^†^**	0.118	**0.488 ^†^**	**0.351 ^†^**	**0.556 ^†^**	**0.446 ^†^**	0.188
EPWORTH Daily Sleep; Total Score	**0.274 ^†^**	**0.350^†^**	0.235	**0.401 ^†^**	−0.033	**0.313 ^†^**	**0.406 ^†^**	0.123	**0.392 ^†^**	**−0.379 ^†^**	**0.431 ^†^**	**0.595 ^†^**	0.122	**0.542 ^†^**	**−0.385 ^†^**
Pittsburgh Sleep Quality Index; Total Score	**0.362 ^†^**	0.066	**0.543 ^†^**	**0.252 ^†^**	0.037	**0.489 ^†^**	0.240	**0.536 ^†^**	**0.354 ^†^**	−0.049	**0.456 ^†^**	0.236	**0.504 ^†^**	**0.332 ^†^**	**0.434 ^†^**
Physical Activity; IPQA	0.076	0.102	0.002	0.095	**0.493 ^†^**	−0.163	−0.115	−0.189	−0.156	**0.379 ^†^**	−0.029	0.003	−0.167	−0.043	**0.284 ^†^**
Walking (meters/week)	0.118	0.072	0.115	0.111	0.132	**−0.380 ^†^**	−0.059	**−0.637 ^†^**	**−0.239 ^†^**	0.096	**0.273 ^†^**	**0.459 ^†^**	−0.019	**0.381 ^†^**	−0.062
Moderate Activity (min-week)	−0.224	−0.093	**−0.328 ^†^**	−0.206	−0.213	**0.350 ^†^**	**0.342 ^†^**	−0.059	**0.295 ^†^**	−0.100	−0.113	**−0.329 ^†^**	0.231	−0.211	0.025
Vigorous Activity (min-week)															
Sexual Activity (women, *n* = 12)	**0.602 ^†^**	**0.644 ^†^**	**0.409 ^†^**	**0.578 ^†^**	**0.256 ^†^**	−0.159	0.101	**−0.544 ^†^**	−0.128	**−0.453 ^†^**	**0.808 ^†^**	**0.842 ^†^**	**0.774 ^†^**	**0.869 ^†^**	**0.276 ^†^**
Desire	**0.662 ^†^**	**0.752 ^†^**	**0.251 ^†^**	**0.579 ^†^**	**0.790 ^†^**	**0.492 ^†^**	**0.593 ^†^**	0.001	**0.452 ^†^**	−0.031	**0.594 ^†^**	**0.580 ^†^**	**0.728 ^†^**	**0.660 ^†^**	0.153
Excitation	0.140	**0.284 ^†^**	−0.206	0.082	**0.628 ^†^**	0.152	0.173	0.015	0.137	**0.518 ^†^**	**−0.246 ^†^**	−0.102	**−0.371 ^†^**	−0.199	**0.413 ^†^**
Lubrication	−0.129	−0.073	−0.018	−0.062	**−0.296 ^†^**	**0.640 ^†^**	**0.747 ^†^**	**0.292 ^†^**	**0.684 ^†^**	−0.114	**−0.527 ^†^**	**−0.363 ^†^**	**−0.721 ^†^**	**−0.505 ^†^**	**−0.378 ^†^**
Orgasmic	**0.767 ^†^**	**0.679 ^†^**	**0.726 ^†^**	**0.747 ^†^**	**0.537 ^†^**	**0.691 ^†^**	**0.466 ^†^**	**0.801 ^†^**	**0.660 ^†^**	**0.448 ^†^**	**0.692 ^†^**	**0.593 ^†^**	**0.664 ^†^**	**0.653 ^†^**	**0.609 ^†^**
Penetration	**0.537 ^†^**	**0.575 ^†^**	0.193	**0.446 ^†^**	**0.661 ^†^**	**0.625 ^†^**	**0.477 ^†^**	**0.492 ^†^**	**0.557 ^†^**	0.093	**0.662 ^†^**	**0.574 ^†^**	**0.459 ^†^**	**0.570^†^**	**0.320 ^†^**
Previous Anxiety															
Sexual activity (men, *n* = 8)	**0.549 ^†^**	**0.406 ^†^**	0.071	**0.512 ^†^**	0.093	**0.383 ^†^**	**0.362 ^†^**	**−0.734 ^†^**	**0.252 ^†^**	−0.236	**0.380 ^†^**	**0.368^†^**	0.088	**0.351 ^†^**	0.142
Overall Sexual Functioning	−0.129	**−0.299 ^†^**	**0.831 ^†^**	0.006	−0.083	0.119	0.123	**0.868 ^†^**	**0.255 ^†^**	**0.283 ^†^**	0.072	0.194	−0.096	0.181	0.212
Sexual Function Distress	**0.573 ^†^**	**0.593 ^†^**	**−0.682 ^†^**	**0.412 ^†^**	**0.263 ^†^**	0.027	0.037	**−0.900 ^†^**	−0.099	−0.198	0.010	−0.110	0.194	−0.091	−0.119
Change Sexual Functioning	−0.054	−0.176	**0.815^†^**	0.136	**−0.389 ^†^**	0.119	0.123	**0.868 ^†^**	**0.255 ^†^**	**0.283 ^†^**	**−0.249 ^†^**	−0.226	**−0.322 ^†^**	−0.237	**0.656 ^†^**
Masturbation	0.076	0.102	0.002	0.095	**0.493 ^†^**	−0.163	−0.115	−0.189	−0.156	**0.379 ^†^**	−0.029	0.003	−0.167	−0.043	**0.284 ^†^**

*Note.*^†^ Bold: effect size into the moderate (|*r*| > 0.24) to good range (|*r*| > 0.30). 1. Baseline, 2. Maximum ketosis, 3. Reduced ketosis, 4. Endpoint.

## Supplementary Materials

The following are available in the http://www.mdpi.com/2072-6643/10/10/1348/s1, Table S1: Evolution of the questionnaires in the 4 assessments through the intervention (*n* = 20).
