# Supplementary material for: Effect of A Very Low-Calorie Ketogenic Diet on Food and Alcohol Cravings, Physical and Sexual Activity, Sleep Disturbances, and Quality of Life in Obese Patients
Source: Nutrients. 2018 Sep 21;10(10):1348. doi: 10.3390/nu10101348 (PMC6213862; doi:10.3390/nu10101348)
Supplement: Supplementary file 1 [file nutrients-10-01348-s001.pdf]

Effect of A Very Low-Calorie Ketogenic Diet on Food and Alcohol Cravings, Physical and Sexual Activity, Sleep Disturbances, and Quality of Life in Obese Patients

Ana I Castro [1,2,†], Diego Gomez-Arbelaez [1,3,†], Ana B Crujeiras [1,2,†], Roser Granero [2,4,] ,

Zaida Aguera [2,5], Susana Jimenez-Murcia [2,5], Ignacio Sajoux [6], Patricio Lopez-Jaramillo [7], Fernando Fernandez-Aranda [2,5,‡] and Felipe F Casanueva [1,2,*,‡]

[1] Division of Endocrinology, Department of Medicine, Molecular and Cellular Endocrinology Area, Complejo Hospitalario Universitario de Santiago (CHUS), Instituto de Investigación Sanitaria de Santiago (IDIS), and Santiago de Compostela University (USC), Santiago de Compostela (15706) , Spain; anaisabel0121@gmail.com (A.I.C.); diedgomez@gmail.com (D.G-A.); anabelecrujeiras@hotmail.com (A.B.C.); endocrine@usc.es (F.F.C)

[2] CIBER de Fisiopatologia de la Obesidad y Nutricion (CIBERobn), Instituto Salud Carlos III; Madrid (28029) Spain

[3] Faculty of Health Sciences, University of Santander (UDES), Bucaramanga (680003), Colombia

[4] Department of Psychobiology and Methodology, Autonomous University of Barcelona, Barcelona (08193), Spain; Roser.Granero@uab.cat

[5] Department of Psychiatry, Bellvitge University Hospital-IDIBELL, (08908), Spain; zaguera@bellvitgehospital.cat (Z.A.); sjimenez@bellvitgehospital.cat (S.J-M.); ffernandez@bellvitgehospital.cat (F.F-A.)

[6] Medical Department Pronokal, Pronokal Group, Barcelona (08009) Spain; Ignacio.S@pronokal.com

[7] Center for Research in Metabolic Syndrome, Prediabetes and Diabetes, Fundacion Oftalmologica de Santander (FOSCAL), Floridablanca (681004) Colombia; jplopezj@gmail.com

† These authors contributed equally to this work and should be considered co-first authors.

‡ Both authors contributed equally to this work and should be considered co-main authors.

* Correspondence: endocrine@usc.es; Tel.: +34-981-955-069

Table S1. *Evolution of the questionnaires in the 4 assessments through the intervention (n=20)*

	Assessment 1		Assessment 2		Assessment 3		Assessment 4		Trends			Ass.1 vs 2		Ass.1 vs 3		Ass.1 vs 4		Ass.2 vs 3		Ass.2 vs 4		Ass.3 vs 4	
	Mean	SD	Mean	SD	Mean	SD	Mean	SD	Linear	Quadr.	Cubic	*p*	|*d*|	*p*	|*d*|	*p*	|*d*|	*p*	|*d*|	*p*	|*d*|	*p*	|*d*|
Food Craving Questionnaire - Trait																							
Positive reinforcement	16.17	3.91	14.95	3.72	12.11	3.25	12.63	3.48	**.004***	.256	.151	.329	0.32	**.004***	**1.13†**	**.014***	**0.95†**	**.006***	**0.81†**	**.022***	**0.64†**	.621	0.15
Negative reinforcement	8.33	3.22	7.37	2.77	6.53	2.52	6.00	2.40	**.007***	.606	.977	.276	0.32	**.033***	**0.62†**	**.019***	**0.82†**	.105	0.32	**.007***	**0.53†**	.395	0.22
Intentions to eat	8.56	3.11	6.26	2.79	5.68	2.56	6.32	2.87	**.014***	**.008***	.825	**.028***	**0.78†**	**.004***	**1.01†**	**.025***	**0.75†**	.179	0.22	.756	0.02	.269	0.24
Cue-dependent eating	15.00	3.58	10.11	4.53	9.63	3.24	10.32	3.30	**<.001***	**.001***	.296	**.001***	**1.20†**	**<.001***	**1.57†**	**<.001***	**1.36†**	.541	0.12	.898	0.05	.386	0.21
Thoughts-preoccupations food	16.78	6.92	13.32	5.56	12.05	5.79	12.47	5.06	**.006***	**.034***	.871	**.031***	**0.55†**	**.007***	**0.74†**	**.008***	**0.71†**	.085	0.22	.200	0.16	.540	0.08
Feelings of hunger	13.94	3.75	11.11	3.40	9.68	3.13	10.11	2.77	**<.001***	**.005***	.649	**.010***	**0.79†**	**<.001***	**1.23†**	**.001***	**1.17†**	**.003***	0.44	.021	0.32	.331	0.15
Lack of control	20.39	5.47	12.95	4.68	11.16	4.79	12.79	4.25	**<.001***	**<.001***	.489	**<.001***	**1.46†**	**<.001***	**1.80†**	**<.001***	**1.55†**	.120	0.38	.654	0.04	.074	0.36
Negative affect	12.50	5.31	9.05	3.85	8.95	4.16	9.21	3.44	**.003***	.056	.094	**.011***	**0.74†**	**.017***	**0.75†**	**.002***	**0.74†**	.923	0.02	.815	0.04	.770	0.07
Guilty feelings	10.44	3.91	7.05	3.41	6.26	2.90	7.00	2.75	**.004***	**.001***	.604	**.003***	**0.92†**	**.001***	**1.21†**	**.003***	**1.02†**	.362	0.25	.935	0.02	.111	0.26
Total trait score	122.11	27.93	92.16	27.78	82.05	27.80	86.84	23.70	**<.001***	**.002***	.709	**.003***	**1.08†**	**<.001***	**1.44†**	**<.001***	**1.36†**	**.030***	0.36	.111	0.21	.240	0.19
Food Craving Questionnaire - State																							
Intense desire to eat	6.33	4.13	5.68	2.89	5.00	2.71	5.26	3.26	.172	.300	.833	.267	0.18	.152	0.38	.178	0.29	.454	0.24	.747	0.14	.633	0.09
Anticipation positive reinforcement	6.83	3.24	6.16	3.06	5.32	2.33	5.47	2.70	.151	.464	.530	.494	0.21	.122	**0.54†**	.202	0.46	.229	0.31	.451	0.24	.712	0.06
Anticipation negative states	6.89	3.08	5.42	2.89	4.47	2.78	5.84	3.08	.264	**.006***	.374	**.044***	**0.49†**	**.015***	**0.82†**	.357	0.34	.177	0.34	.499	0.14	.064	0.47
Preoccupation with food-lack control	7.44	3.13	3.84	1.21	3.89	1.45	4.63	2.09	**.003***	**<.001***	**.002***	**<.001***	**1.52†**	**<.001***	**1.46†**	**.001***	**1.06†**	.863	0.04	.115	0.46	.103	0.41
Craving as physiological state	8.06	3.65	7.16	2.65	7.95	3.50	8.00	3.67	.772	.286	.349	.128	0.28	.782	0.03	.998	0.02	.377	0.25	.195	0.26	.748	0.01
Total state score	35.56	11.99	28.26	10.66	26.63	10.80	29.21	10.86	.096	**.007***	.759	**.005***	**0.64†**	**.023***	**0.78†**	.069	**0.55†**	.587	0.15	.543	0.09	.119	0.24
Food Craving Inventory (FCI, SP)																							
Simple sugars/trans fats	16.68	10.86	8.79	7.79	8.79	7.13	9.21	7.76	**.002***	**.002***	.076	**.001***	**0.84†**	**.001***	**0.86†**	**.001***	**0.79†**	.998	0.00	.744	0.05	.623	0.06
Complex carbohydrates/proteins	12.89	5.35	11.11	6.91	8.00	5.73	8.84	6.18	**.004***	.259	.214	.337	0.29	**.008***	**0.88†**	**.009***	**0.70†**	**.039***	**0.50†**	.145	0.35	.346	0.14
Saturated fat/high calorie content	6.00	3.06	4.79	3.52	3.53	2.72	3.74	2.49	**<.001***	.319	.384	.250	0.37	**.012***	**0.86†**	**.001***	**0.81†**	**.021***	0.40	.126	0.34	.695	0.08
Multidim. Craving Alcohol Scale (MACS)																							
Women ; *n=12*																							
Desire	14.08	4.93	13.33	4.91	13.42	7.54	14.92	8.85	.734	.312	.919	.648	0.15	.662	0.10	.685	0.12	.970	0.01	.555	0.22	.260	0.18
Lack of inhibition	5.17	2.79	5.17	2.79	5.25	2.80	3.50	2.47	.183	.260	.621	1.000	0.00	.944	0.03	.193	**0.63†**	.940	0.03	.069	**0.63†**	.082	**0.66†**
Total score	19.25	5.66	18.50	5.52	18.67	6.91	18.42	8.66	.754	.817	.834	.686	0.13	.653	0.09	.693	0.11	.941	0.03	.975	0.01	.859	0.03
Men; *n=8*																							
Desire	14.00	5.66	11.43	2.51	12.43	3.51	12.29	3.35	.589	.339	.063	.289	**0.59†**	.546	0.33	.489	0.37	.111	0.33	.078	0.29	.604	0.04
Lack of inhibition	5.86	3.08	3.71	3.15	4.86	3.08	5.00	3.11	.670	.271	.090	**.042***	**0.69†**	.420	0.32	.395	0.28	.188	0.37	.329	0.41	.909	0.05
Total score	19.86	6.89	15.14	3.24	17.29	4.42	17.29	5.44	.597	.099	**.047***	**.047***	**0.88†**	.402	0.44	.439	0.41	.145	**0.55†**	.208	**0.52†**	1.000	0.00
Total; n=20																							
Desire	14.05	5.05	12.63	4.21	13.05	6.25	13.95	7.30	.984	.153	.708	.280	0.31	.440	0.18	.945	0.02	.759	0.08	.429	0.22	.285	0.13
Lack of inhibition	5.42	2.83	4.63	2.93	5.11	2.83	4.05	2.74	.156	.831	.274	.416	0.27	.710	0.11	.113	0.46	.527	0.17	.448	0.20	.172	0.38
Total score	19.47	5.96	17.26	4.99	18.16	6.01	18.00	7.48	.545	.224	.330	.141	0.40	.322	0.22	.394	0.22	.549	0.16	.675	0.12	.873	0.02

	Assessment 1		Assessment 2		Assessment 3		Assessment 4		Trends			Ass.1 vs 2		Ass.1 vs 3		Ass.1 vs 4		Ass.2 vs 3		Ass.2 vs 4		Ass.3 vs 4	
	Mean	SD	Mean	SD	Mean	SD	Mean	SD	Linear	Quadr.	Cubic	*p*	|*d*|	*p*	|*d*|	*p*	|*d*|	*p*	|*d*|	*p*	|*d*|	*p*	|*d*|
EPWORTH daily sleep: total score	9.68	5.19	8.05	4.84	7.21	4.43	8.58	4.66	.122	**.010***	.499	.065	0.33	**.010***	**0.51†**	.191	0.22	.212	0.18	.416	0.11	**.036***	0.30
Pittsburgh Sleep Quality Index: total	6.63	4.30	5.79	4.34	5.21	3.03	5.74	3.93	.173	.121	.621	.269	0.19	.062	0.38	.245	0.22	.265	0.15	.918	0.01	.262	0.15
Physical activity; IPQA																							
Walking (meters/week)	805.2	909.3	1603.1	2830.1	1038.7	879.9	1894.1	2336.9	**.050***	.913	.204	.244	0.38	.176	0.26	.062	**0.95†**	.326	0.27	.508	0.13	.089	**0.76†**
Moderate activity (minutes-week)	292.1	969.0	348.3	798.0	437.5	854.1	289.3	623.7	.864	.376	.577	.723	0.06	.457	0.16	.924	0.00	.587	0.11	.615	0.08	.382	0.20
Vigorous activity (minutes-week)	99.5	199.5	148.4	137.6	189.5	463.0	128.2	147.2	.125	.196	.826	**.032***	0.29	.242	0.25	.107	0.16	.671	0.12	.665	0.14	.584	0.18
Sexual activity (women; *n=12*)																							
Desire	5.71	1.98	6.38	1.92	6.67	0.82	6.25	2.25	.564	.341	.703	.530	0.34	.317	**0.63†**	.709	0.25	.580	0.20	1.000	0.06	.636	0.25
Excitation	6.86	1.68	8.00	2.88	8.83	2.40	8.00	2.83	**.043***	.249	.164	.289	**0.50†**	**.010***	**0.99†**	.080	**0.51†**	**.025***	0.31	.341	0.00	.456	0.32
Lubrication	2.29	0.95	3.00	1.20	3.17	0.98	2.50	1.20	**.013***	.203	.661	.235	**0.66†**	**.041***	**0.91†**	**.025**	0.20	.175	0.15	1.000	0.42	.175	**0.61†**
Orgasmic	2.43	1.40	3.38	0.74	2.67	1.51	3.38	0.74	.168	.456	.091	**.034***	**0.85†**	.203	0.16	.135	**0.85†**	.102	**0.60†**	1.000	0.00	.363	**0.60†**
Penetration	4.71	1.60	4.50	1.31	3.83	0.98	3.50	1.77	.312	.296	.451	.849	0.15	.076	**0.66†**	.516	**0.72†**	.141	**0.58†**	.203	**0.64†**	.576	0.23
Previous anxiety	1.29	1.60	0.50	0.53	0.17	0.41	0.38	0.52	.246	.062	1.000	.175	**0.66†**	.121	**0.96†**	.275	**0.76†**	.175	**0.70†**	1.000	0.24	.175	0.45
Sexual activity (men; *n=8*)																							
Overall sexual functioning	13.57	9.71	14.29	10.06	15.43	11.39	15.00	11.08	.455	.321	.647	.564	0.07	.308	0.18	.529	0.14	.425	0.11	.582	0.07	.760	0.04
Sexual function distress	0.29	0.76	0.43	1.13	0.00	0.00	0.00	0.00	.200	.805	.491	.805	0.15	.356	**0.53†**	.356	**0.53†**	.356	**0.53†**	.356	**0.53†**	1.000	0.00
Change sexual functioning	-1.14	1.46	-0.43	1.13	0.29	2.93	0.71	3.95	.350	.569	.793	.283	**0.55†**	.340	**0.62†**	.337	**0.62†**	.454	0.32	.419	0.39	.356	0.12
Masturbation	1.14	1.57	0.86	1.57	0.86	1.57	0.57	0.98	.172	1.000	.172	.356	0.18	.356	0.18	.172	0.44	1.000	0.00	.356	0.22	.356	0.22
Impact of Weight on Quality of Life																							
Physical	30.53	9.60	21.05	9.40	15.84	6.22	14.05	2.95	**<.001***	**.002***	.779	**<.001***	**1.00†**	**<.001***	**1.82†**	**<.001***	**2.32†**	**<.001***	**0.65†**	**.001***	**1.00†**	.084	0.37
Sexual life	8.16	4.63	7.05	4.64	5.21	2.46	4.37	1.01	**.001***	.761	.219	.076	0.24	**<.001***	**0.79†**	**.001***	**1.13†**	**.010***	**0.50†**	**.019***	**0.80†**	.119	0.45
Self-steam	17.84	7.01	12.68	6.47	9.21	3.54	8.32	2.73	**<.001***	**.003***	.734	**<.001***	**0.76†**	**<.001***	**1.55†**	**<.001***	**1.79†**	**.005***	**0.67†**	**.003***	**0.88†**	**.034***	0.28
Social anxiety	6.53	3.56	6.16	3.15	5.37	1.16	5.11	0.32	.080	.607	.232	**.049***	0.11	.059	0.44	.076	**0.56†**	.118	0.33	.128	0.47	.235	0.31
Work area	6.84	3.42	6.21	3.74	4.89	1.63	4.16	0.37	**.004***	.852	.366	.117	0.18	**.001***	**0.73†**	**.003***	**1.10†**	**.044***	**0.51†**	**.029***	**0.77†**	**.049***	**0.62†**
Total score	69.89	22.03	53.16	23.79	40.53	13.31	36.00	5.58	**<.001***	**.005***	.546	**<.001***	**0.73†**	**<.001***	**1.61†**	**<.001***	**2.11†**	**<.001***	**0.66†**	**.002***	**0.99†**	.056	0.44

Note. SD: standard deviation. MD: mean difference. *Bold: significant comparison (.05 level). †Bold: effect size into the moderate (|*d*|>0.50) to good range (|*d*|>0.80). 1. Baseline, 2. Maximum ketosis, 3. Reduced ketosis, 4. Endpoint
